# Lasting *Gammaproteobacteria* profile changes characterized hematological cancer patients who developed oral mucositis following conditioning therapy

**DOI:** 10.1080/20002297.2020.1761135

**Published:** 2020-05-13

**Authors:** Jean-Luc C. Mougeot, Micaela F. Beckman, Craig B. Stevens, Kathryn G. Almon, Darla S. Morton, Inger Von Bültzingslöwen, Michael T. Brennan, Farah Bahrani Mougeot

**Affiliations:** a Department of Oral Medicine, Carolinas Medical Center-Atrium Health, Charlotte, NC, USA; b Institute of Odontology, Sahlgrenska Academy at the University of Gothenburg, Gothenburg, Sweden

**Keywords:** Mucosal immunity, cancer biology, bacteria, hematopoietic stem cell transplantation, transplantation conditioning, hematologic neoplasms

## Abstract

**Background:**

Oral mucositis (OM) is a common side effect of conditioning therapy implemented before hematopoietic stem cell transplantation (HSCT). The role of oral microbiome in OM is not fully elucidated.

**Objective:**

To determine oral microbiome profile changes post-conditioning in HSCT patients who developed moderate OM, or mild to no OM.

**Design:**

Patient groups were: Muc0-1 with OM-score = 0–1 (43 paired samples) and Muc2 with WHO OM-score = 2 (36 paired samples). Bacterial DNA was isolated from oral samples (saliva, swabs of buccal mucosa, tongue, and supragingival plaque) at pre-conditioning (T**_0_**), post-conditioning mucositis onset (T**_Muc_**), and one-year post-conditioning (T**_Year_**). *16S-rRNA* gene next-generation sequencing was used to determine the relative abundance (RA) of >700 oral species. *Alpha*-diversity, *beta*-diversity and linear discriminant analyses (LDA) were performed Muc2 *versus* Muc0-1.

**Results:**

Muc2 oral microbiome *alpha*- and *beta*-diversity differed between T**_0_** and T**_Muc_**. Muc2 *alpha*-diversity and Muc0-1 *beta-*diversity did not differ between T**_0_** and T**_Year_**. T**_0_** to T**_Muc_** LDA scores were significant in Muc2 for *Gammaproteobacteria*. For Muc2 patients, the average RA decreased for *Haemophilus parainfluenza*, a species known as mucosal surfaces protector, but increased for *Escherichia-Shigella* genera.

**Conclusions:**

Post-conditioning OM might contribute to long-term oral microbiome changes affecting *Gammaproteobacteria*, in HSCT patients.

Patients with hematological cancers undergo conditioning therapy prior to hematopoietic stem cell transplantation. A myelosuppressive or myeloablative conditioning regimen provides the anti-cancer effect, while the transplant re-establishes hematopoietic functioning [1,2]. Over 50,000 patients undergo conditioning therapy prior to hematopoietic stem cell transplantation each year [2].

Oral mucositis (OM) is one of the most frequently occurring side effects associated with conditioning therapy. Patients with OM experience damage to the oral mucosa ranging from redness and soreness to ulceration [3,4]. OM grading defined by the World Health Organization (WHO) utilizes a 0 to 4 scale, based on the presence of erythema and soreness (score 1 and above) and/or ulcers (score 2 and above) and the ability for patients to consume solid food (score 2) or liquids only (score 3), or inability to consume any food orally (score 4), due to the painful condition of inflamed oral mucosa [5]. Sonis has developed a five-phase model describing OM pathophysiology [4].

Patients consistently report OM as the most painful and debilitating side effect of cancer treatment [6–8]. The incremental cost of OM-associated hospitalization can be as high as 70,000 USD for patients who develop ulcerative mucositis post-conditioning [9]. The substantial impact of OM, combined with a lack of evidence-based treatment protocols, creates a knowledge gap impacting patient care [10,11].

Various cancers, antibiotic therapy, and myelosuppression by conditioning therapy have been associated with microbial dysbiosis [12]. Nevertheless, little research has explored the relationship between specific microbes and OM. In 2013, Ye et al. analyzed the microbial diversity and richness of pediatric patients with malignancies and found a non-statistically significant increase in the relative abundance of the genera previously associated with OM, such as *Enterococcus, Escherichia, Porphyromonas* and *Pseudomonas* [13]. In agreement with these results, a recent study in chemotherapy patients with solid tumors reported that mucositis severity was positively correlated with three salivary gram-negative bacilli but negatively correlated with 24 commensal species, including *Streptococcus, Actinomyces, Gemella, Granulicatella*, and *Veillonella* genera [14]. Additionally, in patients subjected to conditioning therapy, changes in microbiome diversity and similarity occur, which may increase susceptibility to a proinflammatory state [15,16]. We have also proposed a model integrating the role of the oral microbiome in cancer therapy-induced OM, within the five-step model established by Sonis [4,17].

Approaches aimed at microbial species-level characterization, host/microbe interaction, and functional genomics hold the promise of providing insight into the mechanisms of OM development and the discovery of possible biomarkers of risk assessment.

The purpose of this study was to conduct a species-level longitudinal analysis of the oral microbiome in hematological cancer patients who developed moderate OM compared to those who developed none or mild OM, as a result of conditioning therapy. Next-generation sequencing (NGS) of the V3-V4 hypervariable region of the *16S rRNA* gene was used to identify over 700 oral bacterial species [18,19].

## Materials and methods

### Patient recruitment

Patients diagnosed with hematological cancers scheduled for conditioning therapy were recruited at Carolinas Medical Center–Atrium Health, Charlotte, North Carolina and enrolled in the prospective cohort study: ‘Multicenter Study on the Burden of Illness of Oral Side Effects from Conditioning Therapy Before Stem Cell Transplantation: Ora-stem Study [10]. The study was approved by the Institutional Review Board (IRB) and patients provided informed written consent. Patients with non-cancer-related hematological disorder (*e.g*., immunodeficiency) were excluded. Demographics of the hematological cancer patients (n = 22) used in this study are shown in Table 1, which excludes loss-to-follow up (*e.g*., patient deceased) or the absence of NGS processing due to low sample quality. The patient cohort was stratified for the time points “baseline” [T**_0_**] prior to conditioning, time of ‘oral mucositis occurrence’ [T**_Muc_**], one-year post-transplant [T**_Year_**]), the number of paired samples obtained from four different oral sites, and the patient’s OM status: Muc2 group (moderate OM) and Muc0-1 group (no OM or mild OM) (Table 1).

**Table 1.** Demographics of hematological cancer patients stratified per oral mucositis status post-conditioning therapy.

	T_0_ to T_Muc_^b^		T_0_ to T_Year_^b^	
Criteria Set-All^a^	Muc0-1^c^	Muc2^c^	Muc0-1^c^	Muc2^c^
Patient (M/F)	12 (8/4)	8 (3/5)	8 (5/3)	7 (4/3)
Paired sample	22	15	21	21
Age^d^:				
Median	56.5	56	58	48
Mean	49.8	51.1	51.9	48.3
Standard deviation	18.4	15.5	16.5	13.6
Range	25-76	23-68	25-67	23-63
Ethnicity^e^:				
M: C/AA	4/4	1/2	3/2	2/2
F: C/AA	4/0	3/2	3/0	1/2

^a^Patient cohort (Set-All, n = 22) corresponds to hematological cancer patients undergoing conditioning therapy and having stimulated saliva samples, swabs of buccal mucosa, superficial supragingival plaque, and tongue collected for oral microbiome profiling by next-generation sequencing, pre- and post-conditioning. Demographic data are shown only for patients with successfully collected and NGS processed samples across three time points. There was similar sample type representation between Muc0-1 and Muc2 groups for the T**_0_** to T**_Muc_** time period.

^b^Time points were: ‘T**_0ʹ_** (time at which cancer is present, pre-conditioning); ‘T**_Muc_**’ (time point when patients may have developed OM WHO score 1 to 4, post-conditioning, or time point when patients did not develop OM [OM score 0] at day of transplant, day 7 and day 14 post-conditioning; ‘T**_Year_**’ (time point one-year post-conditioning).

^c^For all three time points combined, the oral mucositis (OM) groups were: (**i**) patients who did not develop OM (score 0) or developed OM with score 1 (MUC**_0-1_**), post-conditioning; (**ii**) patients who developed OM with score 2 (MUC**_2_**), post-conditioning. There were 43 (= 22 + 21) and 36 (= 15 + 21) total paired sample counts T**_0_** to T**_Muc_** and T**_0_** to T**_Year_** combined, for MUC**_0-1_** and MUC**_2_**, respectively. Overall, 114 oral samples forming pairs were collected and sequenced across the three time points. No patient developed a mucositis score of 3 or 4 for this patient cohort.

^d^Average age for T**_0_** to T**_Muc_** and T**_0_** to T**_Year_** patients was 50.9 and 50.2 years old, having standard deviations of 16.7 and 14.8, with ranges of 23 to 76 and 23 to 67 years of age, respectively.

^e^Gender and ethnicity consisted of males (M) and females (F) of Caucasian (C) or African American (AA) ethnicity.

De-identified clinical characteristics of the patient cohort (n = 22) included: (**i**) worst WHO OM scores, (**ii**) hematological stem cell transplant type (autologous (4 Muc0-1 and 2 Muc2 patients)) or allogeneic (9 Muc0-1 and 7 Muc2 patients), (**iii**) hematological cancer diagnoses, paired oral samples (T**_0_** to T**_Muc_** or T**_0_** to T**_Year_)**, and paired for all three time points, (**iv**) whether or not patients had received antibiotic prophylaxis treatment (levofloxacin) within 2 weeks prior to oral sample collection at T**_0_** pre-conditioning (antibiotics treatment before T**_0_** beyond two-weeks possible, but no antibiotics treatment between T**_0_** and T**_Muc_**), and (**v**) chemotherapy with or without total body irradiation (TBI) (Supplementary Table 1).

### Sample collection and processing

Oral samples were collected at least one following dental manipulations (*e.g*., eating, oral hygiene). Stimulated saliva (S) samples, and swab samples from buccal mucosa (B), superficial supragingival plaque (P), and tongue (T) were collected. Samples were obtained at baseline (T**_0_**: one to 8 weeks pre-conditioning), days of likely occurrence of OM (T**_Muc_**: day of transplant (day 0) post-conditioning, day 7 and day 14 post-transplant) and 12 months post-transplant ±30 days (T**_Year_**). Saliva samples were collected at T**_0_** and T**_Year_** only.

Stimulated saliva was collected while chewing unflavored and unsweetened gum base (The Wrigley Company, Mars, Inc., Chicago, IL). The samples (1–2 mL) were centrifuged (2,600 x g; 4°C; 15 min) for pelleting. Other oral samples (B, P, T) were suspended in nuclease-free PBS solution containing 0.04% sodium azide and rotated (2 hat room temperature) to release bacteria. The suspensions were centrifuged (16,000 x g) [20]. All pellets were stored at −80°C.

Bacterial genomic DNA was extracted from oral samples using the modified QIAamp DNA Mini Kit procedure (QIAGEN, Valencia, CA) per manufacturer’s instructions. Human Oral Microbe Identification using Next Generation Sequencing (HOMINGS) was used to identify bacteria at the species and genus levels, and relative abundances were determined as previously described [18,19,21]. Briefly, the amplified *16S rRNA* gene (V3–V4 region) was sequenced using a modified MiSeq *NGS* method (Illumina, Inc., San Diego, CA) [21]. Oral taxa identification and abundance were determined using the ProbeSeq program, in which sequence reads were first matched against ProbeSeq species probes, in a BLAST-type electronic ‘*e*-hybridization’ [18–20]. The number of sequence-reads matched to one probe of 737 probes total (620 ProbeSeq species probes and 117 genus probes) was counted. The relative abundance of ProbeSeq matched genus probes and species probes was determined for each patient sample.

### Bioinformatics analysis

#### Beta-diversity analysis

**Figure 1.**
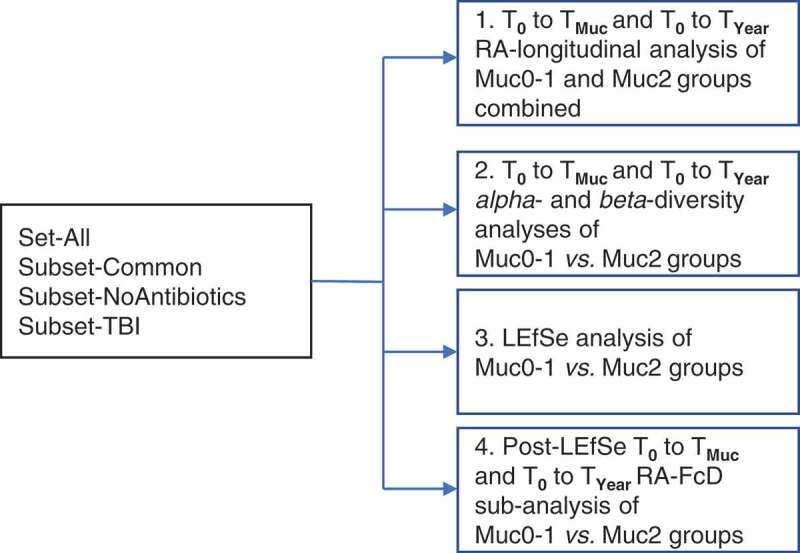
Analytical design for fold changes and changes in beta-diversity of hematological cancer patients undergoing conditioning therapy with and without oral mucositis.

The overall analytical strategy is presented in Figure 1. Groups of patients for time period analyses were Set-All, Subset-Common, Subset-noAntibiotics, and Subset-TBI (Supplementary Table 1). The time period analyses T**_0_** to T**_Muc_** and T**_0_** to T**_Year_** were performed for Muc0-1 and Muc2. For Muc0-1 and Muc2, there were, respectively, 43 and 36 total paired sample counts for T**_0_** to T**_Muc_** and T**_0_** to T**_Year_** combined, *i.e*., overall 114 individual oral samples forming pairs in effect were collected and sequenced. Cross-sectional analyses were performed to compare Muc0-1 and Muc2 at T**_0_**, T**_Muc_** and T**_Year_**. For both OM groups, if the same highest WHO score was present during more than one time point at T**_Muc_**, relative abundance data were averaged. Relative abundance data were squared-root transformed and converted to Bray–Curtis similarity matrices followed by PERMANOVA analyses using a mixed-model with unrestricted permutation of raw data, 9,999 permutations, and type III partial sum of squares, in the PRIMER**_v7_** program (PRIMER-E Ltd., Ivybridge, UK), as previously implemented by our group [20]. Fixed factors in the PERMANOVA longitudinal design were ‘Time’ (T**_0_** to T**_MUC_** and T**_0_** to T**_Year_**) and ‘Site’ (up to four levels: S, B, P and T). In this design, the random factor ‘Treatment’ was coded as a single factor with four possible outcomes (*i.e*., four levels: Antibiotics [yes/no] and TBI [yes/no]) to control for degrees of freedom and was nested into ‘Site’. The random factor ‘Subject’ was nested in ‘Treatment’ and ‘Site’ in the longitudinal analysis but was replaced by ‘Group’ (Muc0-1, Muc2) in the cross-sectional analysis. Monte-Carlo corrected p-values (α = 0.05) were determined and PCoA comparisons of relative abundance sample data of T**_0_** *vs*. T**_MUC_** and T**_0_** *vs*. T**_Year_** for Set-All groups Muc0-1 and Muc2 were carried out in PRIMER**_v7_** to visualize significant comparisons (PERMANOVA) (Supplementary Figure 1a and 1b).

#### Alpha-diversity determination

Shannon and Simpson indices were generated using PRIMER**_v7_**. Wilcoxon signed-rank and Mann–Whitney U tests were used in time period Muc0-1 *vs*. Muc2 comparisons, respectively (α = 0.05), using XLSTAT**_v2016.02.29253_**.

#### Linear discriminant analysis (LDA) effect size (LEfSe)

Taxonomy levels were added manually to ProbeSeq derived datasets for Set-All groups Muc0-1 and Muc2. The tabular text file was formatted to perform LEfSe using online tool Galaxy**_v1.0_** [22]. LEfSe data input consisted of ‘Group’ (Muc0-1and Muc2) for the LEfSe input option ‘Class’ and ‘Patient’ for the LEfSe input option ‘Subject’ in the LEfSe online program Galaxy**_v1.0_** [22]. Using the ‘one-against-all’ strategy for multi-class analysis [23], the factorial Kruskal–Wallis test and pairwise Wilcoxon signed-rank test were set at a Monte-Carlo significance level α = 0.05 to calculate LDA scores. The log LDA score was set at a threshold >0 and used to generate a cladogram representing the hierarchy of all significant biomarkers and a histogram of the top biomarkers, plotted at the genus and species levels [22].

#### Post-LEfSe proteobacteria sub-analysis

Genus and species probes belonging to the *Proteobacteria* phylum (n = 91 probes) were divided into *Gammaproteobacteria* (n = 30) and remaining (n = 61) probes. Longitudinal PERMANOVA analyses T**_0_** to T**_Muc_** and T**_0_** to T**_Year_** were performed for both Muc0-1and Muc2 groups using the species and genera probes for ‘*Gammaproteobacteria’* and ‘all *Proteobacteria’*. PCoA were generated in PRIMER**_v7_**.

Further, the relative abundance fold change difference (RA-FcD), based on the formula [(‘final RA’ minus ‘initial RA’) divided by ‘initial RA’], for each oral site, was determined from the relative abundance data obtained after adding a pseudo-count of +1 to each raw data count, as previously described [20,24]. The average RA-FcD sum per each sample site for all the patients in the Muc0-1 or Muc2 group, based on the total abundance of the 30 *Gammaproteobacteria* probes (not total of 737 probes) per sample, was calculated for the T**_0_** to T**_Muc_** time period. The sample site representation for Muc0-1 and Muc2 groups was similar, *i.e*., 3/2 B, 6/4 P, and 12/8 T samples, respectively. A Fisher’s exact test (α = 0.05) was then performed for each time point in R**_v3.4.3_** [R 25] to determine if the average RA-FcD sum per oral site across time points were significantly different between Muc0-1 and Muc2. Specificity and sensitivity and Receiver Operating Characteristic (ROC) curves were determined [26].

## Results

### Abundance data, species detection, and alpha-diversity

Sequencing reads matched to 737 total probes (620 species and 117 genus probes) for all paired samples from our patient cohort at time points T**_0_**, T**_Muc_** and T**_Year_** (overall total of 114 samples forming pairs collected and sequenced) are summarized in Supplementary Table 2. Unmatched reads were excluded from relative abundance determinations. For all samples with sequencing data available, 397 of 620 species probes and 85 of 117 genus probes had at least one read matched to a genus or species probe. Derived relative abundances were used for analysis of patient subsets Set-All, Subset-Common, Subset-noAntibiotics and Subset-TBI (Figure 1). In the T**_0_** *vs*.T**_Muc_** comparison for Muc0-1, there was no *alpha*-diversity difference for any subsets (Simpson and Shannon) (Set-All result is shown in Supplementary Table 3). However, in the T**_0_** *vs*. T**_Year_** comparison, there was a significant *alpha*-diversity difference for Set-All (Shannon). In the T**_0_** *vs*. T**_Muc_** comparison for Muc2, there was a significant difference in *alpha*-diversity for Set-All (Simpson and Shannon), while there was none T**_0_** *vs*. T**_Year_**.

We also found that the ranges of taxa detected per patient for Muc0-1 and Muc2 groups combined across T**_0_** to T**_Muc_** and T**_0_**-T**_Year_**, were 43.42 to 87.86 for genera and 129.5 to 281.14 (Supplementary Table 3).

### Beta-diversity analyses

The baseline T**_0_** cross-sectional PERMANOVA analyses of Muc0-1 *vs*. Muc2 resulted in no significant *beta*-diversity difference for any patient subsets, suggesting a similar oral microbiome ‘starting point’ (data not shown). There were *beta*-diversity differences for both time periods T**_0_** to T**_MUC_** and T**_0_** to T**_Year_** for Set-All and Subset-noAntibiotics, and Subset-TBI for T**_0_**-T**_MUC_** only, when combining data from Muc0-1 and Muc2 (p < 0.05) (Supplementary Table 4).

In the Set-All T**_0_** toT**_Muc_** comparison, differences in *beta*-diversity changes were noted when Muc0-1 and Muc2 groups were analyzed individually. There were no significant *beta*-diversity changes for Muc0-1 subsets. Although stratification reduces sample size, the Muc2 T**_0_** to T**_Muc_** results suggest that *beta*-diversity changes in relation to moderate OM (*i.e*., p < 0.05 for Set-All, Subset-noAntibiotics, Subset-TBI, marginal p-value for Subset-Common) were possibly related to the absence of antibiotics treatment. Indeed, 7 of 13 Muc0-1 patients received TBI and were treated with antibiotics within two weeks prior to sampling at T**_0_** pre-transplant, compared to a single patient treated with antibiotics among 9 Muc2 patients including 6 treated with TBI for conditioning but not antibiotics within two weeks prior to T**_0_** (Supplementary Table 1). In the T**_0_**-T**_Year_** comparison, for similar numbers of patients and samples, there was no *beta*-diversity change for Set-All in Muc0-1 group, as opposed to Set-All in Muc2 (Table 2). Overall, although Muc0-1 and Muc2 groups had similar patient and sample counts representation, most of the comparisons for the Muc2 group (Set-All and Subsets) were significant or marginally significant, whereas the Muc0-1 comparisons generated only one significant p-value (Set-all T**_0_** *vs*.T**_Muc_**) (Table 2).

**Table 2.** Longitudinal PERMANOVA analyses of separate oral mucositis groups, Muc0-1 and Muc2, based on the PERMANOVA fixed factor ‘Time’.

Muc0-1^a^	# of pts^b^	Paired sample (Ct)^c^	Time period^d^	*p*-value^e^
Set-All	12	22	T**_0_**-T**_Muc_**	0.003
Subset-Common	7	11	T**_0_**-T**_Muc_**	0.579
Subset-NoAntibiotics	5	11	T**_0_**-T**_Muc_**	0.094
Subset-TBI	10	6	T**_0_**-T**_Muc_**	0.355
Set-All	8	21	T**_0_**-T**_Year_**	0.291
Subset-Common	7	11	T**_0_**-T**_Year_**	0.334
Subset-NoAntibiotics	5	14	T**_0_**-T**_Year_**	0.127
Subset-TBI	6	15	T**_0_**-T**_Year_**	0.335
Muc2	# of pts	Paired sample (Ct)	Time period	p-value
Set-All	8	15	T**_0_**-T**_Muc_**	0.017
Subset-Common	6	10	T**_0_**-T**_Muc_**	0.058
Subset-NoAntibiotics	8	15	T**_0_**-T**_Muc_**	0.018
Subset-TBI	6	11	T**_0_**-T**_Muc_**	0.029
Set-All	7	21	T**_0_**-T**_Year_**	0.031
Subset-Common	6	10	T**_0_**-T**_Year_**	0.102
Subset-NoAntibiotics	6	19	T**_0_**-T**_Year_**	0.051
Subset-TBI	4	12	T**_0_**-T**_Year_**	0.156

^a^Patient groups analyzed were designated as Muc0-1 and Muc2 with the first group corresponding to patients with no OM or OM score 1 and the second group representing patients with OM score 2, post-conditioning. Set-All in the Muc0-1 and Muc2 groups consisted of all patients having paired sample data (stimulated saliva, buccal mucosa, superficial supragingival plaque, or tongue) for T**_0_** to T**_Muc_** [T**_0_**-T**_Muc_**] and T**_0_** to T**_Year_** [T**_0_**-T**_Year_**] time periods; ‘Subset-Common’ corresponds to all patients in common (*i.e*., those having matched samples by sample site for all three time periods, n = 21 pairs); ‘Subset-NoAntibiotics’ represents patients who did not receive antibiotics within 2 weeks prior to sampling at T**_0_** pre-transplant; ‘Subset-TBI’ describes patients who received total body irradiation during conditioning.

^b^Number of patients for each patient group.

^c^Total number of paired patient samples for each group. There were 43 (= 22 + 21) and 36 (= 15 + 21) total paired sample counts, considering T**_0_** to T**_Muc_** and T**_0_** to T**_Year_** time periods combined, for Muc0-1 and Muc2, respectively.

^d^Time points were: ‘T**_0ʹ_** (time at which cancer is present, pre-conditioning); ‘T**_Muc_**’ (time point when patients developed OM WHO score 1 to 2, post-conditioning, or time point when patients did not develop OM [OM score 0] at day of transplant (day 0), day 7 and day 14 post-transplant; ‘T**_Year_**’ (time point one-year post-transplant). Analyses were performed for the time periods T**_0_** to T**_Muc_** and T**_0_** to T**_Year_**.

^e^Longitudinal PERMANOVA analyses were performed for Muc0-1 and Muc2 groups separately, based on Bray–Curtis similarity matrices determined from square root transformed relative abundance data derived from screening of all 737 probes comprised of 620 species and 117 genus probes, using PRIMER**_v7_** (PRIMER-E Ltd., Ivybridge, UK). Monte-Carlo corrected p-values for the fixed factor ‘Time’ (α = 0.05) are shown. Significant p-values are highlighted (grey), marginal p-values are underlined.

### LEfSe analysis of Muc2 and Muc0-1 groups

LEfSe [22] identified 54 differential features for Muc0-1 and Muc2 in Set-All consisting of 737 total probes (620 species and 117 genus probes). *Proteobacteria* (log LDA ≈ −0.01) was identified as the leading differential feature between Muc0-1 and Muc2 (Figure 2(a, b)). *Veillonella* made up the largest differential feature for Muc0-1 (log LDA ≈ 0.003). When comparing the list of genera determined by LEfSe to the list representing 10 species/genera with the largest significant RA-FcD changes for T**_0_** to T**_Muc_**, *Abiotrophia, Capnocytophaga, Gemella, Haemophilus, Lactobacillus, Prevotella* and *Veillonella* genera overlap (Figures 2(b) and 3).

**Figure 2.**
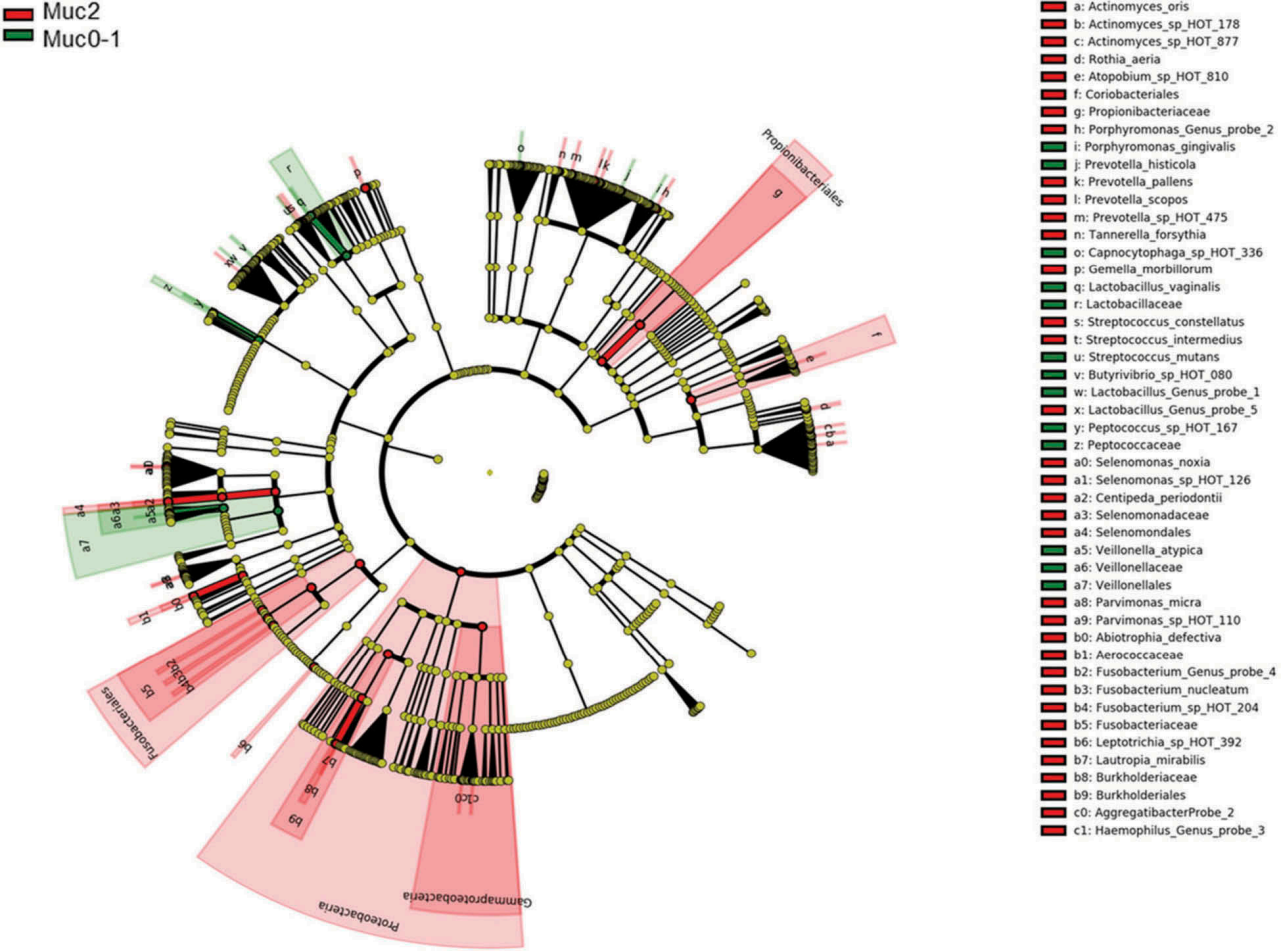
LEfSe results of set-all Muc0-1 and Muc2 groups.

**Figure 2.**
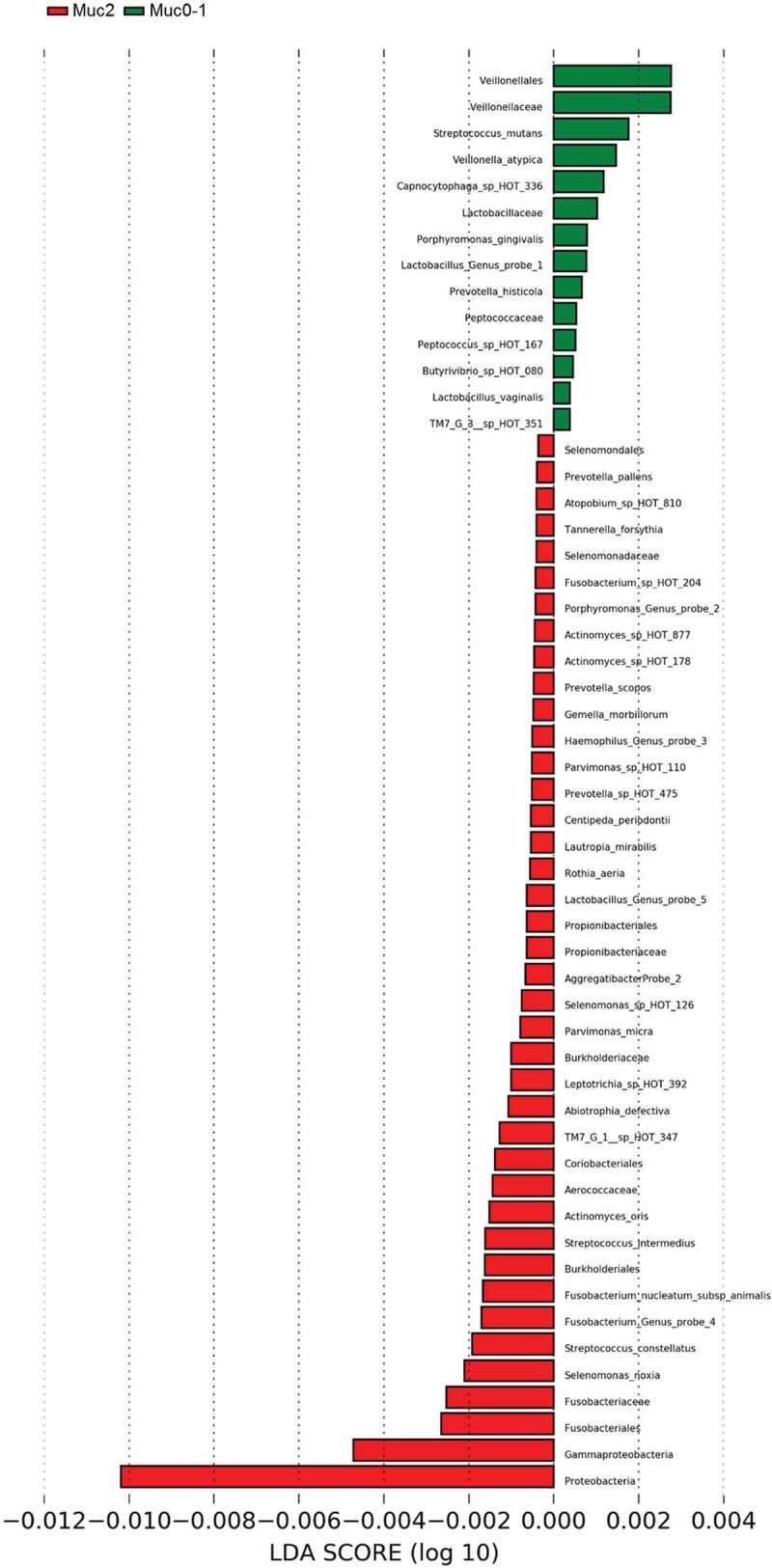
(Continued).

**Figure 3.**
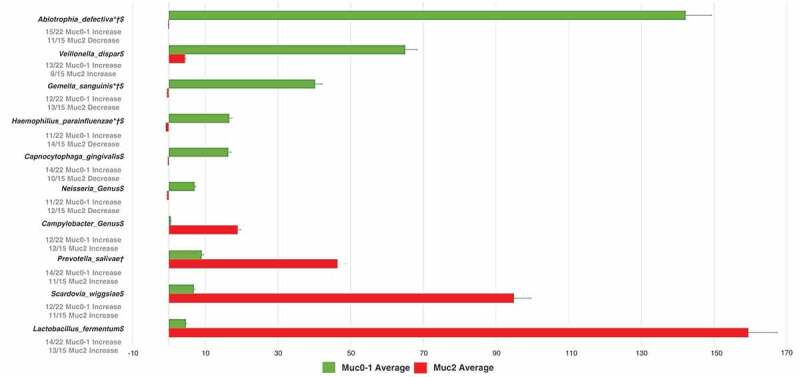
Average fold change (RA-FcD) difference from T_0_ to T_Muc_ distinguishing Muc0-1 from Muc2 hematological cancer patient groups undergoing conditioning therapy.

### *Post-LEfSe analysis of* Proteobacteria

Since the LEfSe results suggested more prominent RA changes occurring in *Proteobacteria*, with *Gammaproteobacteria* representing the largest subgroup, PERMANOVA analyses were performed using corresponding probe data. The T**_0_** *vs*. T**_MUC_** analysis of 91 *Proteobacteria* showed significance for Muc2 (p = 0.0022), but not for Muc0-1 (p = 0.0643), while analysis of T**_0_** *vs*. T**_Year_** did not show significance for Muc2 or Muc0-1 (p > 0.05). The T**_0_** *vs*.T**_MUC_** analysis of the 30 *Gammaproteobacteria* probes resulted in significance for Muc2 (p = 0.0015), but not for Muc0-1 (p = 0.3415). The T**_0_** *vs*.T**_Year_** analysis of 30 *Gammaproteobacteria* probes found Muc2 to be not significant (p = 0.0742) while Muc0-1 was significant (p = 0.0386). PCoA plots for these comparisons are shown in Supplementary Figure 2, *i.e., Proteobacteria* in Figure 2(a, b, c, d), and *Gammaproteobacteria* in Figure 2(e, f, g, h).

In addition, Fishers’ exact test was performed using the average of the sum of RA-FcD per oral sample site across Muc0-1 and Muc2 patients for each of the 30 *Gammaproteobacteria* probes. There was an overall average RA-FcD difference between the Muc0-1 and Muc2 group in the T**_0_** *vs*.T**_MUC_** (p = 4.03e-3) and T**_0_** *vs*. T**_Year_** (p = 1.17e-10) comparisons. Notably, from T**_0_** to T**_MUC_** and T**_0_** to T**_Year_** time periods the relative abundance of *Haemophilus parainfluenza* decreased, on average per sample site, while it increased for *Escherichia* and *Shigella* genera in Muc2 compared to Muc0-1. ROC curves showed that the specificity for the 30 *Gammaproteobacteria* probes was 0.75 regarding T**_0_**-T**_Muc_** RA-FcD changes and 0.724 for the T**_0_** to T**_Year_** comparison (Supplementary Figures 3a and 3b).

## Discussion

This is the first one-year follow-up study investigating oral mucositis-associated microbiome profiles in multiple oral sites of hematological cancer patients undergoing conditioning therapy prior to hematopoietic stem cell transplant. The difference between patients who develop OM with a WHO score of 2 and those who do not (scores 0–1) is the presence of ulcer(s) in the oral mucosa. Therefore, the comparison Muc0-1 *vs*. Muc2 provided the opportunity for better understanding oral microbiome shifts associated with the ‘threshold’ of presence of ulcers in oral cavity. More severe OM (scores of 3 and 4) might involve additional sequential, possibly confounding host responses affecting the oral microbiome. Increased risk for bacteremia or infection may then be associated with the development of pseudomembranes on mucosal ulcerations with additional colonization of bacterial species capable of promoting further inflammation [27]. However, previous studies have positioned the oral microbiome as an OM exacerbator rather than an initiator [4,15]. Here we sought to determine which microbial sub-community may be disrupted, thereby leading to OM exacerbation.

Using V3-V4 *16S rRNA* gene next-generation sequencing along with the ProbeSeq species and genera identification program, we were able to detect at least 482 bacterial species from the 737 species and genus probes (65.4%) for our patient cohort, considering that approximately 700 predominant taxa are represented in oral cavity [28]. Changes in *alpha*-diversity consistently occurred during T**_0_**-T**_MUC_** time period for the Muc2 group (Simpson and Shannon). Conversely, there were no changes in *alpha*-diversity for Muc0-1 for that time period.

The PERMANOVA results (Supplementary Table 4) showed that by analyzing Muc0-1 and Muc2 patients combined, the *beta*-diversity significantly changed for the T**_0_**-T**_MUC_** time period with Set-All (including the AB ‘treatment’ factor as defined earlier) and Subset-noAntibiotics yielding identical p-values. When analyzing Muc0-1 and Muc2 separately (Table 2), there was a significant *beta*-diversity change for both groups for the Set-All subset at T**_Muc_**. Significant *beta*-diversity changes for the T**_0_**-T**_MUC_** time period were more frequent among Muc2 subsets. For this time period, none of the Muc2 patients were exposed to antibiotics treatment close to T**_0_**, while 58% Muc0-1 patients were (Supplementary Table 1, Table 2). However, large well-controlled studies did not demonstrate that antibiotics prophylaxis reduces incidence or severity of OM [11]. Additionally, there was a *beta*-diversity change at T**_Year_** for Muc2, but not for Muc0-1, in Set-All. These results taken together suggest that the oral microbiome community in our patients’ cohort who developed moderate OM did not return to a normal state, in contrast to Muc0-1 patients. Long-term microbial shifts have been associated with an OM history in a patient population with Fanconi Anemia who underwent conditioning therapy [29].

*Proteobacteria* showed the largest linear discriminant analysis score (log LDA = 0.011), potentially explaining why microbial communities did not recover after one-year. LEfSe also showed that many more features distinguish Muc2 compared to Muc0-1. Furthermore, *Gammaproteobacteria* were major contributors to the differences observed between Muc0-1 and Muc2, as shown by PERMANOVA and ROC analyses.

Following the post-LEfSe sub-analysis on the 30 *Gammaproteobacteria* probes, we observed the largest average of RA = FcD sum per oral site ratios for the T**_0_** *vs*.T**_MUC_** time period, between Muc0-1 and Muc2, corresponding to a decrease in Muc2, for *H. parainfluenzae* and the *Acinetobacter* genus, whereas an increase was observed for the *Pseudomonas* genus, *Pseudomonas fluorescens* and *Escherichia* and *Shigella* genus. Of 15 Muc2 oral samples, for T**_0_**-T**_Muc_**, the average RA-FcD [(‘final RA’ minus ‘initial RA’) divided by ‘initial RA’] of *H. parainfluenza* (calculated from 30 probes total abundance, not the 637 probes) decreased in 14 samples (average RA-FcD [SD] = −0.94 [0.12]) and increased in one sample (RA-FcD = +0.57). Of 22 Muc0-1 oral samples, for T**_0_**-T**_Muc_**, the average RA of *H. parainfluenza* decreased in 11 samples (average RA-FcD [SD] = −0.77 [0.26]) and increased in the remaining half (average RA-FcD [SD] = +30.01 [71.85]). In contrast, for this same time period, average RA of *Escherichia* and *Shigella* (genus probe) increased in 10 of 15 samples in Muc2 with an average RA-FcD [SD] = +23.90 [36.41] compared to 14 of 22 samples in Muc0-1 with a significantly lower average RA-FcD [SD] = +3.31 [4.8]. Although reduced, these differences subsisted relatively similar, T**_0_**-T**_Year_**.

Previous studies in patients with sarcoidosis and oral lichen planus have provided evidence that *H. parainfluenza* is protective of mucosal surfaces, especially strains that are strong biofilm producers [30,31]. Although our data indicate a clear trend in this respect, abundance data alone cannot predict risk for OM, since strains of *H. parainfluenza* would need to be identified and functional assays to assess biofilm production capacity be performed.

Considering the low LDA effect sizes in our study, more research with a larger sample size, bacterial strain level identification, and functional analyses related to biofilm production may be required to determine composite microbiome signatures providing adequate diagnostic value to predict OM severity. Indeed, we were unable to differentiate *Escherichia* and *Shigella* species identified by a genus probe and therefore we have no precise knowledge about the potential for invasiveness affecting the oral mucosa.

In addition, while this study focused on the characterization of the oral microbiome profiles in hematopoietic stem cell transplant (HSCT) patients who develop moderate OM compared to patients with OM scores of 0–1, understanding sequential changes occurring in patients who develop severe OM (scores 3–4) would provide additional clinical significance to our study. In particular, one might show that certain microbiome changes for patients who transition from moderate to severe OM would be characteristic of patients who experience the most pain. Furthermore, the absence of salivary flow data at T**_Muc_**, precluded an analysis including such variable, which along with other factors (*e.g*., conditioning regimen; engraftment type) can influence the microbiome composition and activity. Another limitation of the study was the loss of samples’ data, due to patient death or absence of multiple visits, therefore, a larger patient cohort and further collaborative efforts would likely provide more comprehensive insights into OM development in HSCT patients.

In conclusion, based on *beta*-diversity results, hematological cancer patients who develop moderate OM following conditioning therapy experience a lasting change in *Proteobacteria* subcommunities up to one-year post-conditioning. More research is needed to define susceptibility to OM, including investigation of host responses. Oral microbiome profiling and functional analysis could provide new means to prevent or mitigate OM in this compromised patient population.

## Supplementary Material

Supplemental Material
