# Supplementary material for: Lasting Gammaproteobacteria profile changes characterized hematological cancer patients who developed oral mucositis following conditioning therapy
Source: J Oral Microbiol. 2020 May 13;12(1):1761135. doi: 10.1080/20002297.2020.1761135 (PMC7269028; doi:10.1080/20002297.2020.1761135)
Supplement: Supplemental Material [file ZJOM_A_1761135_SM3623.docx]

**Lasting *Gammaproteobacteria* profile changes characterize hematological cancer patients who developed oral mucositis following conditioning therapy**

Jean-Luc C. Mougeot^1*^, Micaela F. Beckman^1^, Darla S. Morton^1^, Inger von Bültzingslöwen^2^, Michael T. Brennan^1^ and Farah Bahrani Mougeot^1^*

**Supplementary Table 1. Summary of clinical metadata of hematological cancer patients undergoing conditioning therapy and paired samples with microbiome NGS data**

| **Set-All**  **Patients^a,c^** | **WHO worst OM Score^b^** | **Subset-No Antibiotics^c,d^** | **Subset**  **TBI^c,e^** | **HSCT type^f^** | **Cancer Diagnosis^g^** | **Paired samples Set-All**  **T_0_-T_Muc_ &**  **T_0_-T_Year_^h^** | **Paired samples Subset-Common^h^** | **T_Muc_ Sample Days^i^** |
| --- | --- | --- | --- | --- | --- | --- | --- | --- |
| **Pt-01** | 1 | No | No | Auto | Myeloma | BPT & BST | BT | 7 / 7 / 7 |
| **Pt-02** | 0 | Yes | Yes | Allo | CML | T & No data |  | 0,7 |
| **Pt-03** | 2 | No | Yes | Allo | ALL | PT & T | T | 0,7 / 0,7 |
| **Pt-04** | 1 | No | Yes | Allo | ALL | PT & ST | T | 7,14 / 14 |
| **Pt-05** | 2 | No | Yes | Allo | ALL | BPT & BPST | BPT | 14 / 14 / 14 |
| **Pt-06** | 2 | No | Yes | Allo | CML | T & BPST | T | 14 |
| **Pt-07** | 0 | Yes | Yes | Allo | MDS | PT & BST | T | 14 / 14 |
| **Pt-08** | 0 | No | Yes | Allo | ALL | PT & PST | PT | 0,7,14 / 0 |
| **Pt-09** | 0 | Yes | Yes | Allo | CML | PT & No data |  | 0,7 / 0 |
| **Pt-10** | 2 | Yes | No | Allo | AML | No data & BS |  |  |
| **Pt-11** | 0 | No | Yes | Allo | AML | BT & BST | BT | 0,7 / 0,7,14 |
| **Pt-12** | 1 | Yes | Yes | Allo | AML | T & ST | T | 0 |
| **Pt-13** | 0 | No | No | Auto | Myeloma | BT & BST | BT | 0,7 / 0,7,14 |
| **Pt-14** | 0 | Yes | Yes | Allo | Lymphoma | PT & No data |  | 0,7,14 / 0,7 |
| **Pt-15** | 2 | No | No | Auto | Myeloma | PT & BPST | PT | 7,14 / 7, 14 |
| **Pt-16** | 2 | No | Yes | Auto | Lymphoma | BPT & No data |  | 7,14 / 14 / 14 |
| **Pt-17** | 0 | No | Yes | Auto | AML | No data & ST |  |  |
| **Pt-18** | 2 | No | No | Allo | Myeloma | PT & PST | PT | 7 / 0 |
| **Pt-19** | 0 | Yes | Yes | Auto | AML | T & No data |  | 0,7,14 |
| **Pt-20** | 2 | No | Yes | Allo | AML | T & PST | T | 0,7 |
| **Pt-21** | 0 | Yes | Yes | Allo | Lymphoma | PT & No data |  | 7,14 / 0,7,14 |
| **Pt-22** | 2 | No | Yes | Allo | MDS | T & No data |  | 7,14 |

**^a^**Hematological cancer patient cohort (N=22) subjected to conditioning therapy prior to hematopoietic stem cell transplant (HSCT) and having microbiome next generation sequencing (NGS) data for a total of 114 samples forming pairs collected across three timepoints, including stimulated saliva (S) samples, and swabs of buccal mucosa (B), superficial supragingival plaque (P), and/or tongue (T).

**^b^**World Health Organization (WHO) worst oral mucositis (OM) score post-conditioning at day of transplant, and day 7 and day 14 post-conditioning. The OM scores obtained for this cohort were 0 (no oral mucosa symptom), 1 (soreness and/or erythema, *i.e.*, mild OM) and 2 (presence of at least one ulcer and ability for solid food consumption, *i.e.*, moderate OM). Patients were stratified in two groups for the overall statistical analysis: Muc0-1 and Muc2 groups.

**^c^**‘Set-All’ represents all patients having paired sample data (stimulated saliva, buccal mucosa, superficial supragingival plaque, or tongue) for T**_0_** to T**_Muc_** and T**_0_** to T**_Year_** time periods; ‘Subset-NoAntibiotics’ represents patients who did not receive antibiotics within two weeks prior to sampling at T**_0_** pre-transplant; ‘Subset-TBI’ describes patients who received total body irradiation during conditioning.

**^d^**Eight of 9 patients with OM score 2 were not treated with antibiotics within two weeks prior to T**_0_**, whereas 5 of 13 patients with OM score 0-1 were not treated with antibiotics within two weeks prior to T**_0_**.

**^e^**Six of 9 patients with OM score 2 and 11 of 13 patients with OM score 0-1 received TBI treatment during conditioning.

**^d,e^**Seven patients with OM score 0-1 received both AB and TBI treatments, whereas no patient with OM score 2 received both treatments.

**^f^**HSCT type corresponds to patients who either received autologous (Auto) or allogeneic stem cell transplants (Allo).

**^g^**Patients were diagnosed with hematological cancers (*i.e.*, non-cancer related immunodeficiency excluded): CML, Chronic Myelogenous Leukemia; ALL, Acute Lymphoblastic Leukemia_;_ MDS, Myelodysplastic Syndrome_;_ AML, Acute Myelogenous Leukemia.

**^h^**All samples (S, B, P, and T) processed and sequenced are described (*i.e.*, loss of samples was related to technical issues and/or patient death, *i.e*., No data). There were, respectively 36 total paired data for patients with moderate OM and 46 for patients with OM score <2, T**_0_** to T**_MUC_** and T**_0_** to T**_Year_** time periods combined, corresponding to a total of 114 individual samples forming pairs collected and sequenced. ‘Subset-Common’ corresponds to all patients in common across time points, *i.e.*, those having matched samples by sample site for T0, T**_Muc_** and T**_Year_** (n=21 paired samples)

**^i^**For both Muc0-1 and Muc2 OM groups, relative abundance data from samples corresponding to same highest WHO score present during more than one timepoint (days 0, 7, and/or 14) at T**_Muc_** were represented as an average of relative abundance data for each sample site. For patients with OM scores of 0, all three days of data have been used (if available), whereas for scores of 1 or 2, only the data of the days where the highest mucositis score was consistent were used. For example, at T**_Muc_**, for **Pt-02** the ‘T’ sample microbiome data of day 0 and 7 were used, while ‘P’ data of days 7 and 14 and ‘T’ data day 14 only, were used for **Pt-04**.

**Supplementary Table 2. 16S Next generation sequencing aggregate raw sequence reads detection data from oral sites of hematological cancer patients, pre- and post-conditioning therapy**

|  | **T_0_-T_Muc_** (n=20) | | | | | | | |
| --- | --- | --- | --- | --- | --- | --- | --- | --- |
| **Descriptive Statistics** | **T_0_ Sp^a^** | **T_0_ G^b^** | **T_0_ U^c^** | **T_0_ TR^d^** | **T_Muc_ Sp^a^** | **T_Muc_ G^b^** | **T_Muc_ U^c^** | **T_Muc_ TR^d^** |
| Total (Ct) | 888013 | 674549 | 366329 | 1928891 | 704136 | 658525 | 342104 | 1704764 |
| Range (Ct) | 1696-78673 | 1214-53967 | 698-42573 | 6932-118673 | 716-56534 | 1049-57753 | 1464-33984 | 9953-102335 |
| Median (Ct) | 21127 | 15443 | 7703 | 53941 | 19175 | 16149 | 5642 | 45844 |
| Mean (Ct) | 24000 | 18231 | 9901 | 52132 | 19031 | 17798 | 9246 | 46075 |
| SD (Ct) | 17146 | 12027 | 8618 | 23301 | 13404 | 12470 | 8592 | 21371 |
| U/TR (%) |  |  | 19.0 |  |  |  | 20.1 |  |
| Range U/TR (%) |  |  | 4.6-45.1 |  |  |  | 5.8-62.9 |  |
|  | **T0-T_Year_** (n=15) | | | | | | | |
| **Descriptive Statistics** | **T_0_ Sp^a^** | **T_0_ G^b^** | **T_0_ U^c^** | **T_0_ TR^d^** | **T_Year_ Sp^a^** | **T_Year_ G^b^** | **T_Year_ U^c^** | **T_Year_ TR^d^** |
| Total (Ct) | 840853 | 684435 | 464716 | 1990004 | 1351961 | 1538601 | 958530 | 3849092 |
| Range (Ct) | 2670-50981 | 993-53967 | 1605-62730 | 9746-75632 | 7371-78079 | 7278-99632 | 5062-81387 | 47617-166252 |
| Median (Ct) | 16912 | 12243 | 8713 | 51998 | 28432 | 27786 | 21175 | 82942 |
| Mean (Ct) | 20020 | 16296 | 11065 | 47381 | 32190 | 36633 | 22822 | 91645 |
| SD (Ct) | 13875 | 13446 | 10875 | 17173 | 14832 | 23460 | 14540 | 28843 |
| U/TR (%) |  |  | 23.4 |  |  |  | 24.9 |  |
| Range U/TR (%) |  |  | 4.8-87.2 |  |  |  | 6.4-53.8 |  |

Next generation sequencing of the 16S rRNA gene V3-V4 hypervariable regions of bacterial genomic DNA was performed on oral samples (stimulated saliva samples, and swab samples of buccal mucosa, superficial supragingival plaque, and tongue) from hematological cancer patients undergoing conditioning therapy (N=22). Samples were collected at T**_0_** pre-conditioning and post-conditioning at T**_Muc_** (time of oral mucositis occurrence) and T**_Year_** (one-year).

Descriptive statistics include the total count (Ct) of abundance, range, median, mean, and standard deviation (SD) for **^a^**species probe matched reads, **^b^**genus probe matched reads, **^c^**unmatched reads, and **^d^**total reads.

In T**_0_** to T**_MUC_** comparison, T**_0_** total species and genus probe matched reads counts (Ct) were 14.7% higher than those of the T**_Muc_** timepoint. The distributions between timepoints of species and genus probe matched reads to total reads by patient sample were not significantly different (p=0.818), as determined by Wilcoxon signed-rank test. This outcome held for the Muc0-1 patients (p=0.650) and for the Muc2 patients (p=0.314, U-test), analyzed separately. T**_0_** total reads (1,928,891) were 13.1% higher than those of T**_Muc_** (1,704,764).

In T**_0_** to T**_Year_** comparison, T**_0_** total species and genus probe matched reads counts were 89.5% lower than those of T**_Year_**. The distributions between timepoints of species and genus probe matched reads to total reads by patient sample was not significantly different (p=0.249). This outcome held for the Muc2 patients (p=0.334), but not for the Muc0-1 patients (p=0.017). T**_Year_** total reads (3,849,092) were 93.4% higher than those of T**_0_** (1,990,004).

**Supplementary Table 3. Simpson and Shannon *alpha*-diversity changes between time points for patients who developed WHO OM score of 2 and those who had scores of 0 and 1.**

| **Set-All**  **[T_0_, T_Muc_, T_Year_]^a^** | **T_0_** | **T_Muc_** |  | **T_0_** | **T_Year_** |
| --- | --- | --- | --- | --- | --- |
| **# of pts**  **Muc0-1/ Muc2^b^** | 12 / 8 | 12 / 8 |  | 8 / 7 | 8 / 7 |
| **# pt samples**  **Muc0-1 / Muc2^c^** | 22 / 15 | 22 / 15 |  | 21 / 21 | 21 / 21 |
| **Average # of species per pt**  **Muc0-1 / Muc2^d^** | 145.67 / 145.88 | 129.5 / 125 |  | 162.13 / 182.43 | 204.63 / 281.14 |
| **Average # of genera per pt**  **Muc0-1 / Muc2^d^** | 43.42 / 49.63 | 44.75 / 51.63 |  | 61.5 / 70.29 | 73.5 / 87.86 |
| ***Simpson Index*** | | | | | |
| **Mean**  **Muc0-1 / Muc2^e^** | 0.932 /0.954 | 0.919 / 0.927 |  | 0.906 / 0.954 | 0.949 / 0.952 |
| **T_0_ to T_Muc /_ T_Year_^f^**  **Muc0-1 pts** | 0.166 |  |  | 0.054 |  |
| **T_0_ to T_Muc /_ T_Year_^f^**  **Muc2 pts** | **0.043** |  |  | 0.798 |  |
| ***Shannon Index*** | | | | | |
| **Mean**  **Muc0-1 / Muc2^e^** | 3.601 / 3.821 | 3.387 / 3.511 |  | 3.310 / 3.889 | 3.667 / 3.864 |
| **T_0_ to T_Muc /_ T_Year_^f^**  **Muc0-1_1_ pts** | 0.179 |  |  | **0.044** |  |
| **T_0_ to T_Muc /_ T_Year_^f^**  **Muc2 pts** | **0.008** |  |  | 0.942 |  |

**^a^**Set-All: HSCT patients (N=22) having next generation sequencing data for stimulated saliva samples and swab samples of buccal mucosa, superficial supragingival plaque, or tongue samples.

**^b^**Number of patients for each patient group.

**^c^**Total number of patient samples for each group.

**^d^**Average number of species/genera detected per patient out of all 737 probes comprised of 620 species and 117 genus probes. Highest and lowest average numbers of taxa (species or genera) detected per patients are shown bold and underlined.

^e^Mean Shannon and Simpson indices for Muc0-1 and Muc2 groups for each time point T**_0_** to T**_Muc_** and T**_Year_**

**^f^**Wilcoxon signed-rank test for the time periods T**_0_** to T**_Muc_** and T**_0_** to T**_Year_**: **^b^**(**i**) T**_0_**, time at which cancer is present, but pre-conditioning; **^b^**(**ii**) T**_Muc_**, time at which patients developed oral mucositis (OM) following conditioning therapy; time at which patients did not develop OM the day of transplant, and day 7 and day 14 post-conditioning; **^b^**(**iii**) T**_Year_**, one-year post-conditioning. Significant p-values in the T**_0_** to T**_Muc_** and T**_0_** to T**_Year_** *alpha*-diversity analyses (Shannon and Simpson indices) are highlighted (grey).

**Supplementary Table 4. Hematological cancer patient subsets, paired samples and longitudinal PERMANOVA analyses regardless of OM development post-conditioning**

| **Pt subsets^a^** | **Timepoint comparison^b^** | **Pt count (M/F)^c^** | **% M/F^d^** | | **Paired samples^e^** | **PERMANOVA time^f^** |  |
| --- | --- | --- | --- | --- | --- | --- | --- |
| **Set-All** | T**_0_**-T**_Muc_** | 20 (11/9) | 55/45 | 37 | | 0.004 | |
| **Set-All** | T**_0_**-T**_Year_** | 15 (9/6) | 60/40 | 42 | | 0.007 | |
| **Subset-Common** | T**_0_**-T**_Muc_** | 13 (8/5) | 62/38 | 21 | | 0.086 | |
| **Subset-Common** | T**_0_**-T**_Year_** | 13 (8/5) | 62/38 | 21 | | 0.157 | |
| **Subset-noAntibiotics** | T**_0_**-T**_Muc_** | 13 (6/7) | 46/54 | 26 | | 0.004 | |
| **Subset-noAntibiotics** | T**_0_**-T**_Year_** | 11 (6/5) | 55/45 | 33 | | 0.007 | |
| **Subset-TBI** | T**_0_**-T**_Muc_** | 16 (9/7) | 56/44 | 28 | | 0.036 | |
| **Subset-TBI** | T**_0_**-T**_Year_** | 10 (6/4) | 60/40 | 27 | | 0.167 | |
|  |  |  |  |  | |  | |

**^a^**Patient subsets analyzed consisted of **(i)** ‘Set-All’ representing all patients having paired sample data (stimulated saliva, buccal mucosa, superficial supragingival plaque, or tongue) for T**_0_** to T**_Muc_** and T**_0_** to T**_Year_** time periods, **(ii)** ‘Subset-Common’ corresponding to all patients in common, *i.e.*, those having matched samples by sample site for all three timepoints, **(iii)** ‘Subset-noAntibiotics’ representing patients who did not receive antibiotics within two weeks prior to sampling at T**_0_** pre-transplant, and **(iv)** ‘Subset-TBI’, *i.e.*, patients who received total body irradiation during pre-conditioning.

**^b^**Timepoints were: ‘T**_0_**’ (time at which cancer is present, pre-conditioning); ‘T**_Muc_**’ (timepoint when patients developed OM WHO score 1 to 4, post-conditioning, or timepoint when patients did not develop OM [OM score 0] at day of transplant, day 7 and day 14 post-conditioning; ‘T**_Year_**’ (timepoint one-year post-conditioning).

**^c^** Patient counts, consisting of Male (M) and Female (F) patients, are also described as the **^d^**percentage of total patients.

**^e^** Paired sample count is the total number of paired patient samples per group.

**^f^** Longitudinal PERMANOVA analyses were performed regardless of the OM development (*i.e.*, patients with or without OM combined in each patient subset), based on Bray-Curtis similarity matrices determined from square root transformed relative abundance data derived from screening of all 737 probes comprised of 620 species and 117 genus probes, using PRIMER**_v7_** (PRIMER-E Ltd., Ivybridge, UK). Monte-Carlo corrected p-values for the fixed factor ‘Time’ (α=0.05) are shown. Significant p-values are highlighted (grey).

**Supplementary Figure 1. PCoA of Set-All at T_0_ to T_MUuc_ and T_0_ to T_Year_**

1. **Set-All T_0_ to T_Muc_**
2. **Set-All T_0_ to T_Year_**

**Legend.**

Principal coordinate analysis (PCoA) of longitudinal PERMANOVA (PRIMER**_v7_**) analysis performed on Bray-Curtis dissimilarity matrices determined from square root transformed relative abundance data using PRIMER**_v7_** (PRIMER-E Ltd., Ivybridge, U.K.) of hematological cancer patient subsets and Monte-Carlo corrected p-values (α= 0.05).

Patient subset used consisted of ‘Set-All’ representing all patients having paired sample data (saliva, buccal mucosa, superficial supragingival plaque, or tongue) for **a.** T**_0_** to T**_MUC_** and **b.** T**_0_** to T**_Year_** time periods.

Timepoints were: ‘T**_0_**’ (time at which cancer is present, pre-conditioning); ‘T**_MUC_**’ (timepoint when patients developed OM WHO score 1 to 4, post-conditioning, or timepoint when patients did not develop OM [OM score 0] at day of transplant (day 0), day 7 and day 14 post-transplant; ‘T**_Year_**’ (timepoint one-year post-transplant).

Muc0-1 group represents patients with no OM or OM score 1 and Muc2 group represents patients with OM score 2, post-conditioning.

**Supplementary Figure 2. PCoA and nMDS of Set-All, T_0_ to T_Muc_ and T_0_ to T_Year,_ for *Proteobacteria* (91 probes) and**

***Gammaproteobacteria* (30 probes)**

***Proteobacteria***

1. **Muc2 PCoA T_0_ to T_Year_**

**a. Muc2 PCoA T_0_ to T_Muc_**


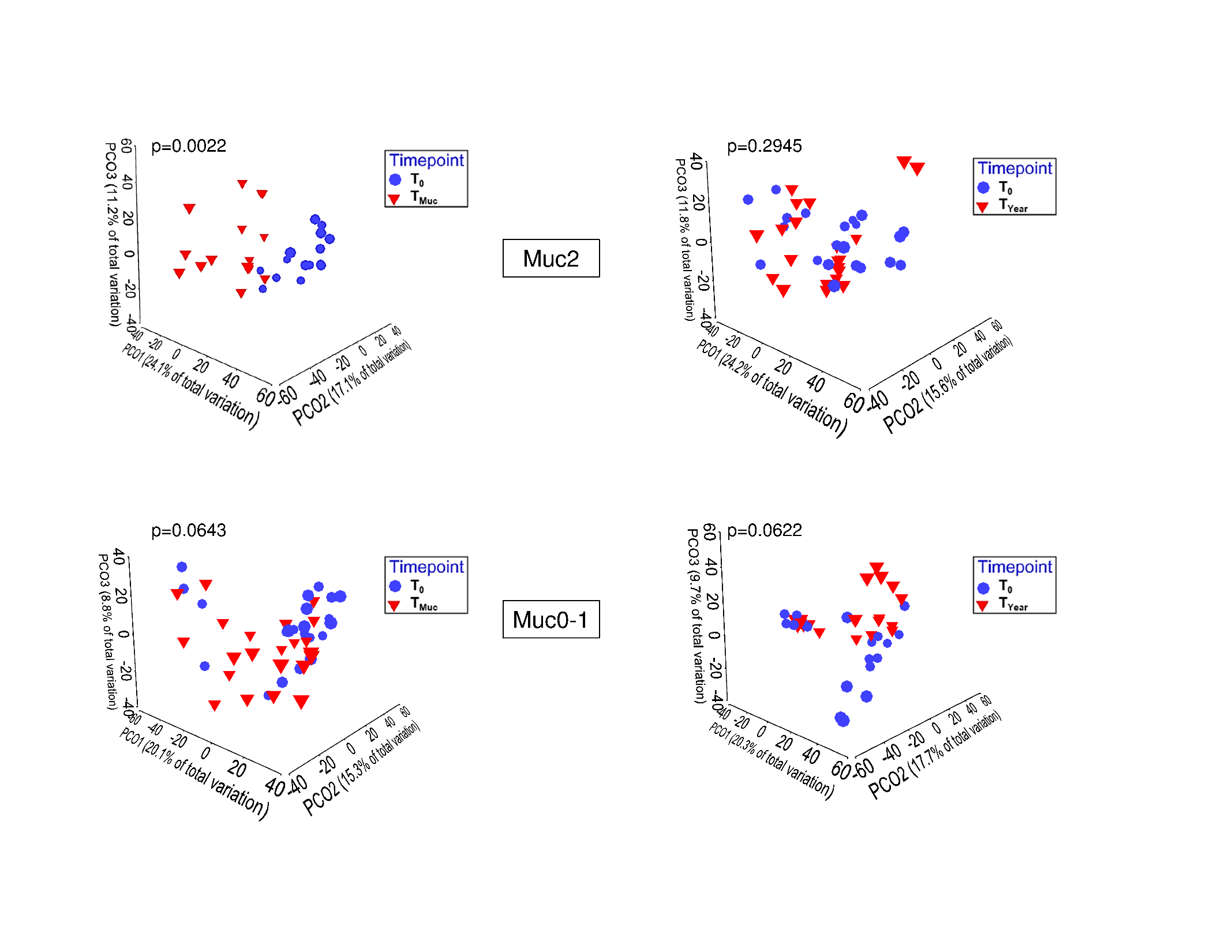


1. **Muc0-1 PCoA T_0_ to T_Year_**

**c. Muc0-1 PCoA T_0_ to T_Muc_**

***Gammaproteobacteria***

1. **Muc2 PCoA T_0_ to T_Year_**

**e. Muc2 PCoA T_0_ to T_Muc_**

1. **Muc0-1 PCoA T_0_ to T_Year_**

**g. Muc0-1 PCoA T_0_ to T_Muc_**


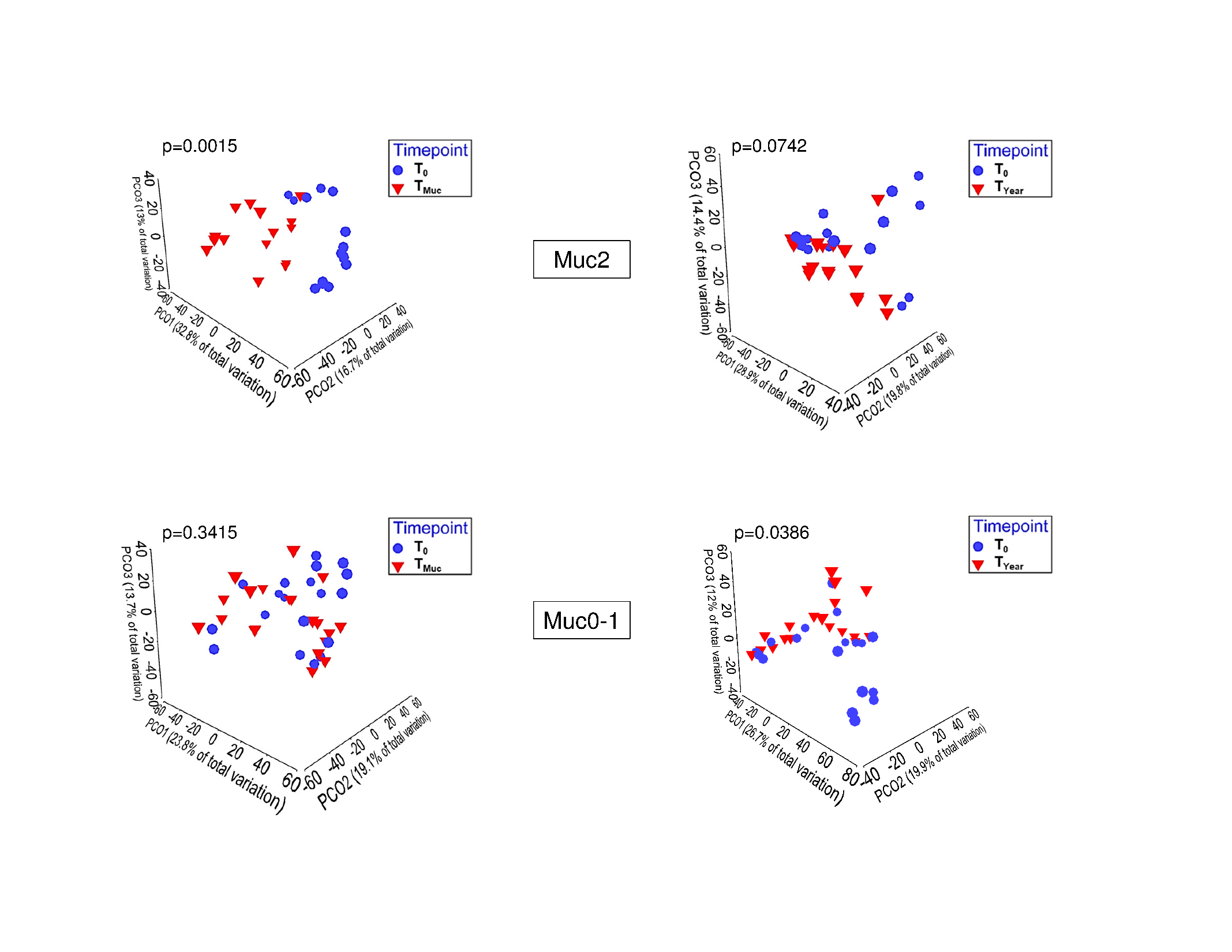


**Legend.**

**a, b, e, and f.** Principal coordinate analysis (PCoA) and **c, d, e, and h.** non-metric multidimensional (nMDS) scaling of longitudinal PERMANOVA (PRIMER**_v7_**) analyses performed on Bray-Curtis dissimilarity matrices determined from square root transformed relative abundance data using PRIMER**_v7_** (PRIMER-E Ltd., Ivybridge, U.K.) of hematological cancer patient subsets and Monte-Carlo corrected p-values (α= 0.05).

Patient subset used consisted of ‘Set-All’ representing all patients having paired sample data (saliva, buccal mucosa, superficial supragingival plaque, or tongue) for T**_0_** to T**_Muc_** and T**_0_** to T**_Year_** time periods split into two groups: **1)** **a, b, c, and d.** ‘***Proteobacteria****’* (n=91 probes) and **2)** **e, f, g, and h.** ‘***Gammaproteobacteria****’* (n=30 probes) based on a total of 737 probes with 620 species probes and 117 genus probes.

Timepoints were: ‘T**_0_**’ (time at which cancer is present, pre-conditioning); ‘T**_Muc_**’ (timepoint when patients developed OM WHO score 1 to 4, post-conditioning, or timepoint when patients did not develop OM [OM score 0] at day of transplant, day 7 and day 14 post-conditioning; ‘T**_Year_**’ (timepoint one-year post-conditioning).

Muc0-1 group represents patients with no OM or OM score 1 and Muc2 group represents patients with OM score 2, post-conditioning.

**Supplementary Figure 3. Determination of sensitivity and specificity based on *Gammaproteobacteria* species and genus probes (n=30) distinguishing Muc0-1 from Muc2 groups of hematological cancer patients undergoing conditioning therapy (Set-All)**

1. **T_0_ to T_Muc_**


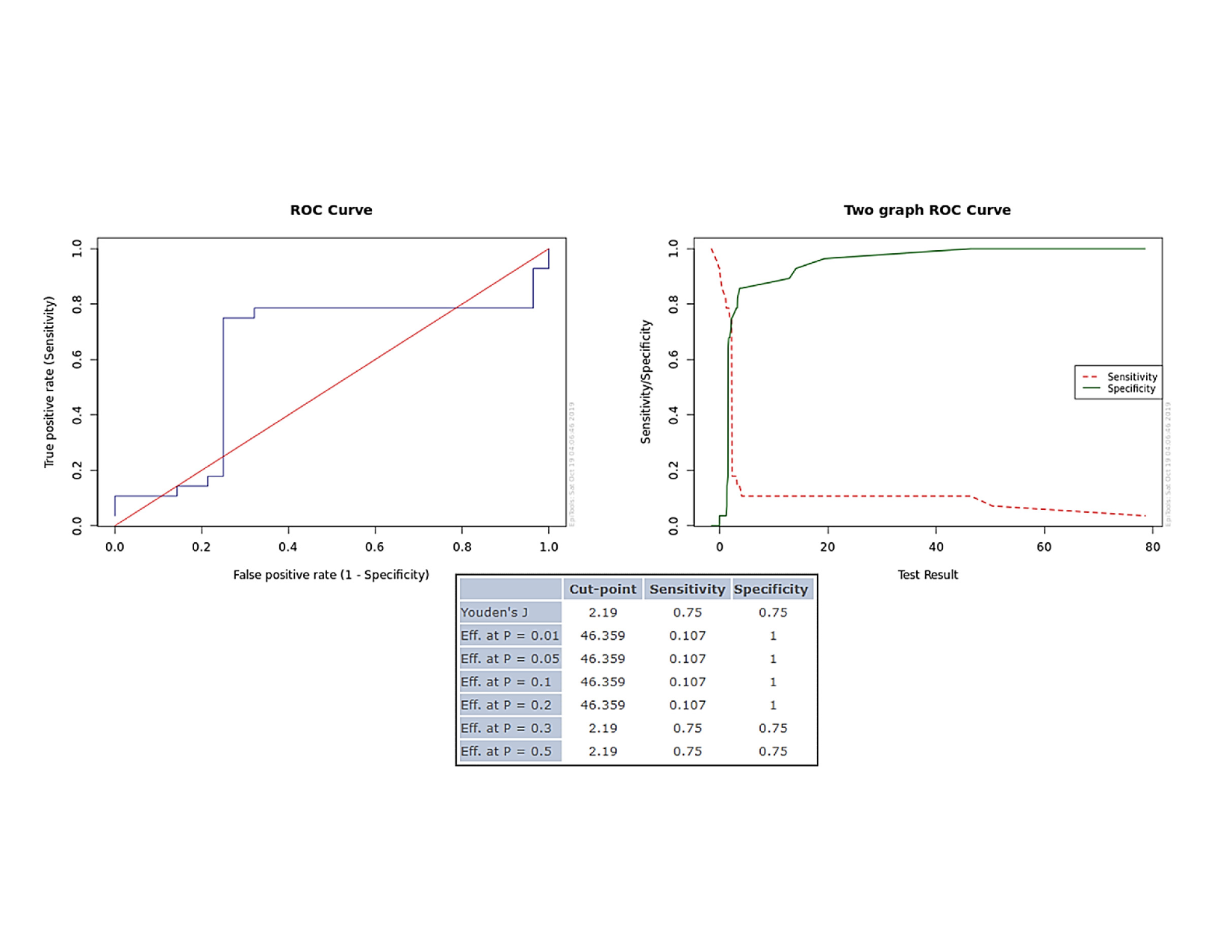


1. **T_0_ to T_Year_**

**
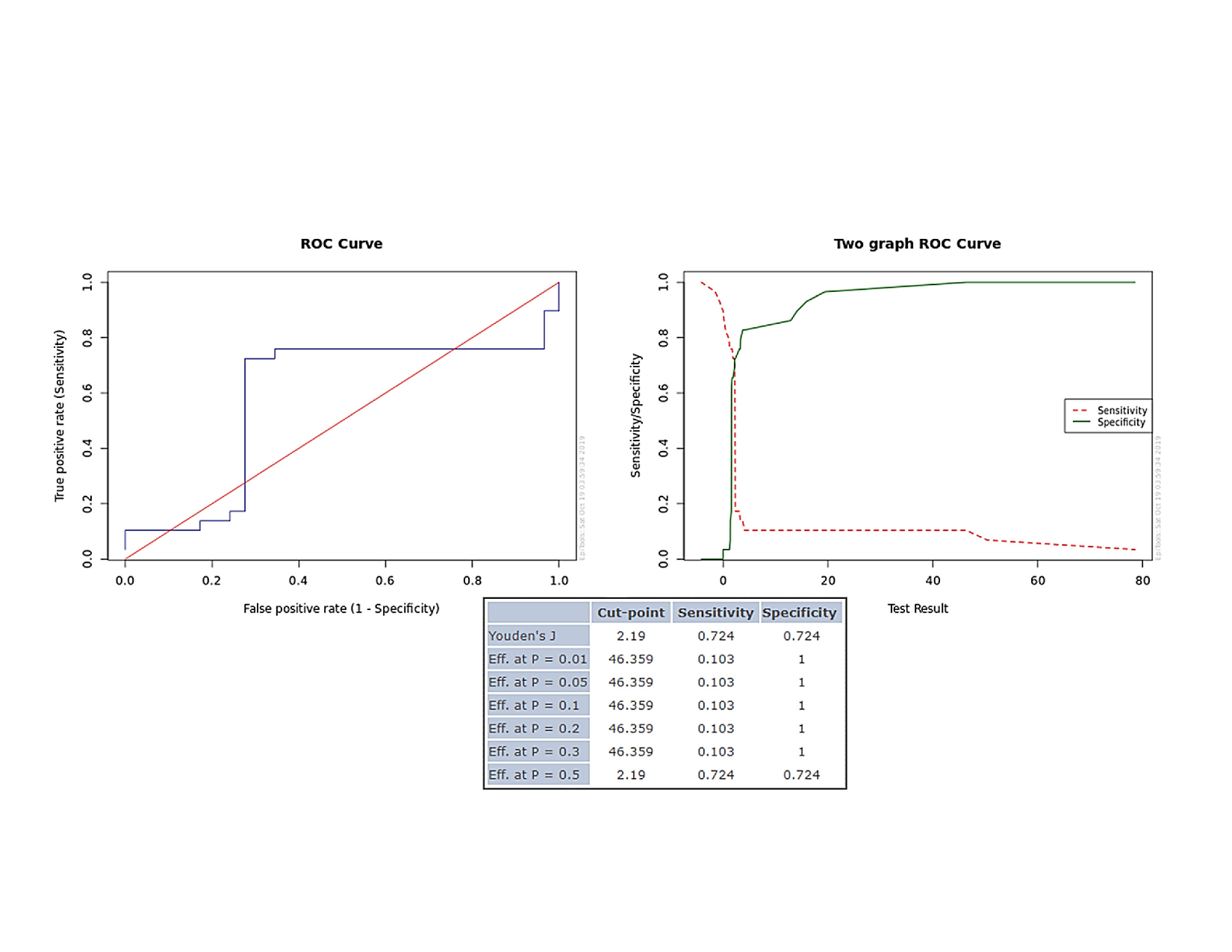
**

**Legend.**

Receiver operating characteristic (ROC) curves for the sensitivity and specificity using *Gammaproteobacteria* probes (n=30) at two time periods **a.** T**_0_** to T**_Year_** and **b.** T**_0_** to T**_Muc_** distinguishing Muc0-1 from Muc2 patients. The curves were generated by using summed average RA-FcD per oral site sample (stimulated saliva and swabs of buccal mucosa, superficial supragingival plaque and tongue).

Timepoints were: ‘T**_0_**’ (time at which cancer is present, pre-conditioning); ‘T**_Muc_**’ (timepoint when patients developed OM WHO score 1 to 4, post-conditioning, or timepoint when patients did not develop OM [OM score 0] at day of transplant (day 0), day 7 and day 14 post-transplant; ‘T**_Year_**’ (timepoint one-year post-transplant).

Muc0-1 group represents patients with no OM or OM score 1 and Muc2 group represents patients with OM score 2, post-conditioning.
